# A Novel NCI-H69AR Drug-Resistant Small-Cell Lung Cancer Mini-Tumor Model for Anti-Cancer Treatment Screening

**DOI:** 10.3390/cells12151980

**Published:** 2023-07-31

**Authors:** Alandi van Niekerk, Krzysztof Wrzesinski, Dewald Steyn, Chrisna Gouws

**Affiliations:** 1 Centre of Excellence for Pharmaceutical Sciences (Pharmacen™), North-West University, Potchefstroom 2520, South Africa; asvniekerk1@gmail.com (A.v.N.); kwr@celvivo.com (K.W.); dewald.steyn@nwu.ac.za (D.S.); 2 CelVivo ApS, 5491 Blommenslyst, Denmark

**Keywords:** cancer modeling, drug resistance, functional spheroid, rotating bioreactors, small-cell lung cancer, three-dimensional cell culture

## Abstract

Small-cell lung cancer is a fast-growing carcinoma with a poor prognosis and a high level of relapse due to multi-drug resistance (MDR). Genetic mutations that lead to the overexpression of efflux transporter proteins can contribute to MDR. In vitro cancer models play a tremendous role in chemotherapy development and the screening of possible anti-cancer molecules. Low-cost and simple in vitro models are normally used. Traditional two-dimensional (2D) models have numerous shortcomings when considering the physiological resemblance of an in vivo setting. Three-dimensional (3D) models aim to bridge the gap between conventional 2D models and the in vivo setting. Some of the advantages of functional 3D spheroids include better representation of the in vivo physiology and tumor characteristics when compared to traditional 2D cultures. During this study, an NCI-H69AR drug-resistant mini-tumor model (MRP1 hyperexpressive) was developed by making use of a rotating clinostat bioreactor system (ClinoStar^®^; CelVivo ApS, Odense, Denmark). Spheroid growth and viability were assessed over a 25-day period to determine the ideal experimental period with mature and metabolically stable constructs. The applicability of this model for anti-cancer research was evaluated through treatment with irinotecan, paclitaxel and cisplatin for 96 h, followed by a 96 h recovery period. Parameters measured included planar surface area measurements, estimated glucose consumption, soluble protein content, intracellular adenosine triphosphate levels, extracellular adenylate kinase levels, histology and efflux transporter gene expression. The established functional spheroid model proved viable and stable during the treatment period, with retained relative hyperexpression of the *MRP1* efflux transporter gene but increased expression of the *P-gp* transporter gene compared to the cells cultured in 2D. As expected, treatment with the abovementioned anti-cancer drugs at clinical doses (100 mg/m^2^ irinotecan, 80 mg/m^2^ paclitaxel and 75 mg/m^2^ cisplatin) had minimal impact on the drug-resistant mini-tumors, and the functional spheroid models were able to recover following the removal of treatment.

## 1. Introduction

According to the GLOBOCAN results of 2020, female breast cancer is the most common type of cancer, followed by lung cancer [1,2]. As far as cancer-related deaths are concerned, lung cancer is a major cause, accounting for 1.8 million deaths per annum (18%) [1,3,4]. Lung cancer can be divided into two broad histological subtypes, namely, non-small-cell lung cancer (NSCLC) and small-cell lung cancer (SCLC) [5]. NSCLC is responsible for 85% of cases, whereas SCLC is responsible for 15%. However, SCLC is known as the graveyard for drug development due to the failure of over 60 possible drug therapies; drug development for the treatment of SCLC has remained stagnant for the last 30 years [6,7]. Due to the rapid doubling time, high growth fraction and natural tendency to metastasize to the brain, liver and bone early in the disease course, the 5-year survival rate when diagnosed with SCLC is 10–20%; thus, this is the most deadly subtype of lung cancer [6,8,9]. Most (70%) of patients are diagnosed in the extensive stage of the disease due to its rapid growth and early widespread metastasis, causing the overall survival rate to be just 6 months [10]. Patients diagnosed in the limited stage of the disease have an overall survival rate of 15–20 months [9]. Another factor having a negative impact on chemotherapeutic treatment in lung cancer is drug resistance. Some tumors respond to a chemotherapeutic drug but develop resistance after a time or the tumor can be resistant directly after the initial therapy [11]. Drug resistance is the consequence of the overexpression of mostly P-glycoprotein (P-gp) or multi-drug resistant protein (MRP) ATP-binding cassette (ABC) efflux transporters [12,13]. The overexpression of these transporters leads to increased drug efflux and decreases the intracellular accumulation of chemotherapeutic agents [11,14].

Irinotecan is a chemotherapeutic drug with a multistep and complex mechanism of action. Inhibition of the topoisomerase 1 enzyme in the DNA results in cytotoxic protein-linked DNA breaks [15]. Poor selectivity between normal and cancerous cells causes serious side effects in the gastrointestinal tract (nausea, vomiting and diarrhea) as well as a drastic effect on bone marrow (neutropenia) [16]. Topoisomerase plays a tremendous role in the transcription, replication and repair of DNA strands. When topoisomerase 1 is inhibited, DNA replication cannot occur, double-strand DNA breaks arise and irreversible cell cycle arrest takes place. G2 cell cycle delay/arrest is initiated and DNA damage is transferred to the S-phase checkpoint mechanism. In higher concentrations of irinotecan, cells in the non-S-phase can also be damaged, initiated by transcription-mediated DNA damage, leading to apoptosis [17]. In cases where low levels of carboxylesterase are present in the tissue, irinotecan cannot be converted into its more active form (SN-38) and drug sensitivity is reduced [17,18]. Another major concern in irinotecan drug resistance is the presence of ABC transporters in the tissue since irinotecan and SN-38 are actively effluxed by P-gp, MRP1 and MRP2 efflux pumps [17,18,19].

Paclitaxel is a taxane chemotherapeutic drug used to treat numerous major cancers, which include metastatic breast, lung, prostate, ovarian, pancreatic and cervical cancers [20]. Paclitaxel induces the breaking of the cancer nucleus into manifold micronuclei through a physical force caused by the paclitaxel-induced rigid microtubules [21]. Regardless of the fantastic results that have been observed, a big concern is the development of drug resistance. Some possible drug resistance mechanisms have been identified, but the major mechanism has not been identified as of yet [22]. Although paclitaxel is a known P-gp substrate, the presence of MRP1 also leads to reduced levels of the drug in lung cancer cells [23,24].

Cisplatin is a platinum-based chemotherapeutic drug used to treat a broad range of cancer types, which include blood, bladder, breast, cervical, esophageal, sarcoma, testicular, lung, head and neck, and ovarian cancers [25,26]. The mechanisms of action of cisplatin are based on the breakage of the DNA helix into single strands when interfering with the purine bases of the DNA. When cisplatin enters the cytoplasm of a cell, chloride is displaced, affecting proteins and nucleic acid, causing the blockage of cell division, and leading to cell death [25,27]. A total of 70–90% of resistance towards cisplatin as a chemotherapeutic drug is based on the reduced accumulation of the drug. ABC transporters have an enormous effect, but another factor is reduced levels of the solute carrier, CTR1, a copper membrane transporter. This is due to the genetic knockout of the transporter caused by cisplatin [25,28].

Only 5% of cancer drugs tested in phase I of clinical trials reach the market; thus, there is a significant need for the development of advanced screening models that mimic disease conditions with high physiological relevance to help eliminate drugs lacking efficacy earlier in the process [29]. The most frequently used models for drug screening are cell monolayers (two-dimensional; 2D). These models have numerous shortcomings that lead to the development of more complex three-dimensional (3D) cell culture models. The complexity of the cell structure, fast growth rate, unnatural morphology, molecular differences, different responses to administrated drugs and lack of a tumor microenvironment are some of the downfalls of 2D cell models that may cause false positive outcomes [30,31,32]. However, 3D cell models can better mimic the tumor microenvironment, and the spatial architecture of 3D models represents tumor growth in the human body and allows cell–cell interaction. These complex models can also develop nutrient, signaling and oxygen factor gradients. Gene expression profiles obtained in 3D models are comparable to in vivo tumors [33].

The four main classes that 3D cell models can be divided into are anchorage-independent models, anchorage-dependent models, microfluidic devices and 3D bioprinting models. The anchorage-independent models include low-adhesion plates, hanging drop methods, magnetic levitation, spinner bioreactors and rotational culture systems (clinostats). The anchorage-dependent methods include natural scaffolds, synthetic scaffolds and hydrogels [34]. During this study, a clinostat bioreactor system was used (CelVivo). These ClinoReactors™ form spheroids in a simulated microgravity environment. The clinostat-based rotating wall vessels concept was developed by NASA in 1992, making it possible for spheroids to form in a low-shear-stress environment [35]. CelVivo ClinoReactors™ consist of two chambers that are divided by a dialysis membrane. The outer chamber plays a role in gas exchange and humidification, while the inside chamber contains the cells and growth medium. These ClinoReactors™ are placed in a rotating system and rotation prevents cells from adhering to the surface of vessels [35,36]. This dynamic system makes it possible for nutrient and gas exchange and waste removal from within the spheroids, creating a controlled environment. Long-term culturing, mass production, and easy medium exchange is made possible by this method [31,37]. High yields of spheroid formation are possible and, although variability in spheroid size is usually a drawback of rotating wall vessels [38], this is rarely the case when using ClinoReactors™.

The objective of this study was to establish a new fit-for-purpose functional NCI-H69AR spheroid model and to characterize the growth and viability of these drug-resistant SCLC mini-tumors over time. The reactivity of the model to treatment was further evaluated by administrating the standard chemotherapeutic drugs irinotecan, paclitaxel and cisplatin to qualify the model and confirm its suitability for further use as a cancer screening tool for potential anti-cancer treatments specifically targeting efflux-based MDR.

## 2. Materials and Methods

### 2.1. Two-Dimensional Culturing of NCI-H69AR Cells

The NCI-H69AR cells (purchased from The American Type Culture Collection (ATCC), #CRL11351™)(LGC Limited, Middlesex, United Kingdom) were cultured using standard culturing conditions. The growth medium, a Roswell Park Memorial Institute (RPMI 1640) medium (Gibco, Thermo Fisher Scientific, Johannesburg, South Africa), was supplemented with 20% fetal bovine serum (FBS) (Gibco, Thermo Fisher Scientific), 1% non-essential amino acids (NEAA) (Lonza; Whitehead Scientific [Pty] Ltd., Cape Town, South Africa), 1% penicillin/streptomycin (10,000 U of each/mL, Lonza; Whitehead Scientific [Pty] Ltd.) and 2 mM L-glutamine (200 mM, Lonza; Whitehead Scientific [Pty] Ltd.). Cells were incubated in an ESCO CelCulture CO_2_ incubator (Esco Technologies [Pty] Ltd., Centurion, South Africa) at 37 °C with 5% CO_2_ and 95% humidified air, with the growth medium exchanged every second day. Subculturing of the cells was initiated upon reaching 80–90% confluence by trypsinization using 0.25% Trypsin-Versene (EDTA) (Lonza; Whitehead Scientific [Pty] Ltd.) [39].

### 2.2. Spheroid Formation and Maintenance

Prior to use, the ClinoReactors™ (CelVivo ApS, Odense, Denmark) culture chambers were prefilled with a growth medium and incubated in the ClinoStar™ system (CelVivo ApS) at 16 rpm to equilibrate (5% CO_2_, 37 °C) for 24 h [40].

Spheroids were formed using the SphericalPlate 5D system (Kugelmeier Ltd., Erlenbach, Switzerland). Wells were washed and prefilled with 500 µL growth medium containing 3 µg/mL L-ascorbic acid (Merck, Sigma-Aldrich, Johannesburg, South Africa) per well. Ascorbic acid plays a role in the folding and deposition of collagen proteins, impacting the extracellular matrix (ECM). A better ECM production improved the structure formation and overall well-being of the spheroids [41]. The SphericalPlate 5D plate was then centrifuged at 1218× *g* for 5 min to remove air bubbles. The wells were seeded with a single-cell suspension containing 750,000 cells per well to yield 1000 cells/microwell, followed by centrifugation at 30× *g* for 30 s and then 140× *g* for 5 min. Incubation for 24 h at 37 °C followed to allow self-aggregation and spheroid formation. The spheroids were removed from the microwells by gently pipetting them with fresh growth medium containing ascorbic acid and they were then transferred to a Petri dish. These detached spheroids were inspected under a light microscope to determine their quality as well as their roundness. Oddly shaped spheroids were removed and the spheroids were then transferred to the equilibrated ClinoReactors™ filled to 10 mL with a fresh growth medium containing 3 µg/mL L-ascorbic acid (day 1 in culturing). The total amount of spheroids obtained per well were placed into a ClinoReactor™ and cultured for 7 days. After 7 days, the spheroids in each ClinoReactor™ were sorted and 180 spheroids were placed into each ClinoReactor™ during the characterization analysis. The ClinoReactors™ containing the spheroids were placed in the ClinoStar™ system at an initial rotation speed of 9.8 rpm. Optimal growth conditions were achieved by exchanging the growth medium every 48 h and adjusting the rotation speed to compensate for spheroid growth.

### 2.3. Characterization of the NCI-H69AR Spheroid Model

The NCI-H69AR spheroids were evaluated to determine the optimal experimental window and observe spheroid viability and growth. The parameters measured included soluble protein content, intracellular adenosine triphosphate (ATP) levels, approximate glucose consumption, planimetric measurements of sampled spheroids and extracellular adenylate kinase (AK) levels. Two biological groups, consisting of two technical replicates (four ClinoReactors™ in total) each, were prepared for characterization on day 7 by transferring 180 spheroids to each ClinoReactor™. This was also the first day of sampling. Spheroids were sampled every second day from alternating biological replicates for a period of 25 days. For example, on day 9, spheroids were sampled from ClinoReactors™ three and four and on day 11, spheroids were sampled from ClinoReactors™ one and two. On day 16, the number of spheroids per ClinoReactor™ were reduced to 140 due to pH levels dropping below 7, and the growth medium was exchanged every 24 h from thereon. On day 19, the number of spheroids were further reduced to 120 spheroids, but the pH remained below 7. Characterization continued to day 25. Each spheroid was viewed as an individual entity that matured and adapted in a self-sustaining manner. Considering this, both biological replicates (sample groups) and technical replicates (number of spheroids/samples) were used. Three spheroid samples for each assay were removed from two technical replicates in a biological replicate, alternating the biological replicates each sampling day. Therefore, six samples were sampled in total from two technical replicates in a biological replicate each sampling day, alternating the biological groups. A total of six spheroids (three spheroids from each technical replicate) were sampled for intracellular ATP assay as well as the soluble protein assay. A total of 200 µL spent medium was sampled for the extracellular AK assay and the rest of the spent media was used to determine the pH and glucose content. The glucose content and pH were measured in duplicate.

#### 2.3.1. Planimetry

The planar surface area of each spheroid sampled was determined from the photomicrographs (4× magnification) taken of spheroids sampled for the Bradford soluble protein assay as well as the intracellular adenosine triphosphate cell viability assay by making use of a Nikon Eclipse TS100 inverted light microscope (Nikon Instruments, Tokyo, Japan) and a DFK 72AUC02 USB 2.0 color industrial camera (The Imaging Source, Bremen, Germany) at 4× magnification. The images were analyzed by making use of the ImageJ software V1.53t (Java2HTML), using the freehand drawing measuring tool to mark the ‘shadow’ area boundaries of the spheroids to determine the planar surface area (µm^2^).

#### 2.3.2. The Bradford Soluble Protein Assay

The Bradford soluble protein assay was used to measure the soluble protein content in each sampled spheroid. The spheroids were sampled into a Costar^®^ flat bottom 96-well plate (Corning Inc., Ascendis Medical, Johannesburg, South Africa). The sampled spheroids were washed with phosphate-buffered saline (PBS) (Hyclone; Separations, Johannesburg, South Africa) and then covered with 150 µL PBS each. Lysis buffers (10 µL) were added to each sample and lysed by pipetting. Protein samples had to be diluted 1:1 on day 13, 1:2 on day 15, 1:3 on day 17, 1:4 on days 19 and 21, 1:5 on day 23, and 1:6 on day 25. PBS was added to each well containing the samples to a final volume of 160 µL. The Bio-Rad protein assay dye reagent concentration (Lasec SA [Pty] Ltd., Midrand, South Africa) was added (40 µL) and mixed vigorously. The plate was centrifuged at 1218× *g* for 2 min, ensuring all air bubbles were removed. Absorbance was measured with a Spectramax^®^ Paradigm plate reader (Paradigm Multi-Mode Detection Platform; Molecular Devices; Separations) at 595 nm. Quantification of the absorbance measurements was done relative to a BSA standard (Lasec SA [Pty] Ltd.) to determine the soluble protein content (µg) of each spheroid [41].

#### 2.3.3. Intracellular Adenosine Triphosphate Cell Viability Assay

The intracellular ATP levels were determined with a CellTiter-Glo^®^ luminescent cell viability assay (Promega, Anatech Instruments [Pty] Ltd., Johannesburg, South Africa). One spheroid was sampled per well, and six spheroids were sampled on each sampling day. The growth medium was replaced with 100 µL PBS and 100 µL CellTiter-Glo^®^ luminescent lysis buffer per well. Spheroids were disrupted by vigorous pipetting [42]. The plate was covered and incubated for 40 min. Air bubbles were removed by centrifugation for 2 min at 1218× *g.* Luminescence values were measured with a Spectramax^®^ Paradigm plate reader with one kinetic window, five measurement cycles with 0.3 s measurement interval time and 2 s delay per measurement, and an additional 0.5 s delay per position change was used. The data were quantified relative to a known ATP disodium salt hydrate standard (Merck, Sigma-Aldrich). Samples were normalized relative to the soluble protein content per spheroid on each sampling day.

#### 2.3.4. Extracellular Adenylate Kinase Cell Death Assay

Spent medium was collected from each experimental group (200 µL) and centrifuged at 140× *g* for 5 min. Subsequently, 160 µL of the supernatant was removed and transferred into a new microcentrifuge tube and centrifuged at 19,480× *g* for 15 min. The supernatant (140 µL) was again transferred into a new microcentrifuge tube, flash-frozen with liquid nitrogen and then stored at −80 °C.

All samples were equilibrated to 22 °C. The samples were loaded in triplicate (20 µL per well) into a black, clear bottom 96-well plate (Corning Inc., Ascendis medical). A dead cell standard was prepared beforehand by treating NCI-H69AR cells (5.59 × 10^5^ cells/mL in RPMI 1640) with a Cyto-Tox Glo™ digitonin lysis buffer (Promega, Anatech Instruments [Pty] Ltd.). The dead cell standard was treated under the same conditions as the other samples and diluted with a heat-treated medium to create a standard curve. The ToxiLight^®^ detection reagent (Lonza, Whitehead Scientific [Pty] Ltd.) (100 µL) was added to each well and mixed. The plate was covered and incubated for 20 min on a compact rocker. The Spectramax^®^ Paradigm plate reader was used to determine the luminescent values of the samples. The resulting values of the samples were quantified relative to the dead cell standard. These values were multiplied by 10 to determine the number of dead cells per 10 mL ClinoReactor™ and normalized relative to the total soluble protein.

#### 2.3.5. Approximate Glucose Consumption

The growth medium was collected to determine the approximate glucose consumption before each medium exchange. Glucose content was measured with a OneTouch Select^®^ blood glucose monitoring system (LifeScan Europe, Zug, Switzerland) and OneTouch Select^®^ blood glucose test strips (LifeScan Europe). The spent medium was loaded onto the test strips (10 µL) to determine the glucose concentrations (mmol/L) in triplicate. Prior to being added to the ClinoReactor™, the glucose concentration of the fresh medium was also measured in triplicate. The value obtained was used as the maximum initial glucose content present in a ClinoReactor™ (23.01 mmol/L). The glucose content of the spent medium was deducted from that of the fresh medium to determine the approximate glucose consumption. This was divided by the total soluble protein present per ClinoReactor™ to determine the approximal glucose consumption per protein (µg) [43].

#### 2.3.6. qRT-PCR Analysis of the Samples

Two spheroid samples were placed in a microcentrifuge tube for each sampling group on day 13. A sample of NCI-H69AR cells cultured in 2D, as well as the NCI-H69V cell line, was also collected. A sample of the NHI-H69V cell line (purchased from the European Collection of Authenticated Cell Cultures [ECACC], Cat# 91091803)(Sigma-Aldrich) cultured as spheroids collected on day 14 of culturing was also used for analysis [41]. These samples were flash-frozen with liquid nitrogen and kept at −80 °C until use. Samples were thawed at room temperature and RNA was extracted with the PureLink™ RNA Mini Kit (Thermo Fisher Scientific) according to the manufacturer’s guidelines. Spheroids were lysed with a lysis buffer containing 1% 2-mercaptoethanol. One volume of ethanol was added to each volume of cell homogenate and vortexed thoroughly. The content was transferred to the spin cartridge and centrifuged at 12,000× *g* for 15 s. The flow-through was discarded. Wash buffer I was added to the spin cartridge and wash buffer II containing ethanol and centrifuged. The flow-through was discarded and the spin cartridge was transferred to a recovery tube. RNase-free water was added to the spin cartridge and incubated for 1 min followed by centrifugation to elute the RNA from the membrane of the spin cartridge. Extracted RNA was quantified on a NanoDrop™ One/OneC UV-Vis spectrophotometer (Thermo Fisher Scientific, Wilmington, DE, USA) at a UV absorbance value of 260 nm. Complementary deoxyribonucleic acid (cDNA) was synthesized using 2 µg of the total RNA and a High-Capacity cDNA reverse transcription kit (Thermo Fisher Scientific) containing an RT buffer, dNTP Mix, RT random primers and MultiScribe™ reverse transcriptase. The prepared content was placed onto a thermal cycler for 10 min at 25 °C, 37 °C for 120 min and 85 °C for 5 min and then cooled down to 4 °C until removal. Real-time PCR was conducted using TaqMan™ Fast Advance Master Mix (Thermo Fisher Scientific) as described in the manufacturer’s guidelines on a C1000Touch™ Thermal Cycler with a 96-Well Fast Reaction Module (Bio-Rad, Lasec SA [Pty] Ltd.). FAM-labeled TaqMan™ Gene Expression assays were used for the following genes: *P-glycoprotein* (*P-gp*) (ABCB1-Hs00184500_m1), *Glyceraldehyde 3-phosphate dehydrogenase* (*GAPDH*) (Hs99999905_m1), *TATA-box binding protein* (*TBP*) (Hs00427620_m1), *Multidrug resistance protein 2* (*MRP2*) (ABCC2-Hs0096089_M1) and *Multidrug resistance protein 1* (*MRP1*) (ABCC1-Hs009905_M1). The housekeeping genes *GAPDH* and *TBP* were used to normalize the data. PCR analyses were performed in triplicate for each sample. UNG incubation was at 50 °C for 2 min and polymerase activation was at 95 °C for 2 min followed by 40 cycles of denature at 95 °C for 1 s and anneal/extend 60 °C for 20 s. Threshold cycle (Ct) values and data were analyzed by making use of Bio-Rad CFX Maestro Software v1.1 (Bio-Rad CFX Maestro. Ink). Relative gene expression was analyzed by making use of the 2-∆∆*Ct* method, as described by Livak et al. [44].

#### 2.3.7. pH Measurements

The pH of the spent growth medium collected from each ClinoReactor™ was measured on each sampling day with an Orion Star A111 pH meter (Thermo Fischer Scientific).

#### 2.3.8. Histological Analysis

On days 9, 13 and 17, spheroids were sampled and fixated in 10% buffered formalin for 7 days. A 10× phosphate buffer solution (Gibco™, Thermo Fisher Scientific) was used as a buffer for the formalin preparation. The spheroids were dehydrated with ethanol at increasing concentrations. The spheroids were paraffin-embedded, cut into 5 µm sections and adhered to Thermo Scientific™ SuperFrost Plus™ adhesion slides (Thermo Fisher Scientific). The slides were stained with hematoxylin-eosin (HE) and alcian blue stain (Sigma-Aldrich) after deparaffination and rehydration to study the morphology of the spheroids.

### 2.4. Evaluation of the NHI-H69AR Spheroid Model for Anticancer Treatment Screening

Reactivity of the NHI-H69AR to treatment was evaluated with three standard chemotherapeutic drugs, cisplatin (European Pharmacopoeia reference standard) (Sigma-Aldrich), paclitaxel from *Taxus breviflolia*, ≥95% powder (Sigma-Aldrich) and irinotecan hydrochloride (Sigma-Aldrich). Setup and maintenance of the ClinoReactors™ were performed in the same manner as that for characterization. On day 7 of spheroid culturing, 150 spheroids were placed into each ClinoReactor™ (two ClinoReactors™ per treatment group). On day 9, treatment was initiated, and sampling took place every 24 h for a 96 h period, after which treatment was suspended and a recovery period of 96 h followed, with sampling every 48 h. Assays were performed as described in Section 2.3.

The dosages of the three chemotherapeutic drugs were determined by calculating the biomass (soluble protein) of the spheroids in each ClinoReactor™ daily after sampling and before treatment and adjusting the chemotherapeutic dose accordingly. The chemotherapeutic doses were based on the typical clinical dose administrated to patients diagnosed with SCLC, namely, paclitaxel (80 mg/m^2^), irinotecan (100 mg/m^2^) and cisplatin (75 mg/m^2^) [45]. The total body protein in a human is 14–16% of their body mass [46]. Taking this into consideration, the chemotherapeutic drug per protein present in a ClinoReactor™ (calculated from the total soluble protein content) could be determined [47].

### 2.5. Statistical Data Analysis

Data analysis was performed with IBM^®^ SPSS^®^ Statistics 27 v27.0 software (IBM Corporation, Armonk, NY, USA). Data collected were statistically analyzed to determine if there were any statistically significant differences between the control group (untreated) and the experimental groups by making use of a Hierarchical Linear modeling method (HLM) [48]. One-way ANOVA followed by the Tukey B posthoc test for the comparison of multiple groups within the control group was used for the analysis of the characterization data. Differences were considered statistically significant when *p* ≤ 0.05.

A one-way ANOVA study with a Bonferroni posthoc test was conducted to analyze the statistical significance of the real-time PCR data obtained by making use of Bio-Rad CFX Maestro 1.1 (4.1.2433.1219) software. Differences were considered statistically significant when *p* < 0.01.

## 3. Results

### 3.1. Characterization of the NCI-H69AR Spheroid Model

NCI-H69AR spheroids were initiated in a Sphericalplate 5D^®^ (SP5D) low-adhesion plate and then transferred to ClinoReactors™. From day 7, over the course of 25 days, the viability and growth of the spheroids were monitored and analyzed by measuring the soluble protein content, intracellular ATP content, glucose consumption, planimetry and the extracellular AK released.

#### 3.1.1. Planimetry

**Figure 1:**
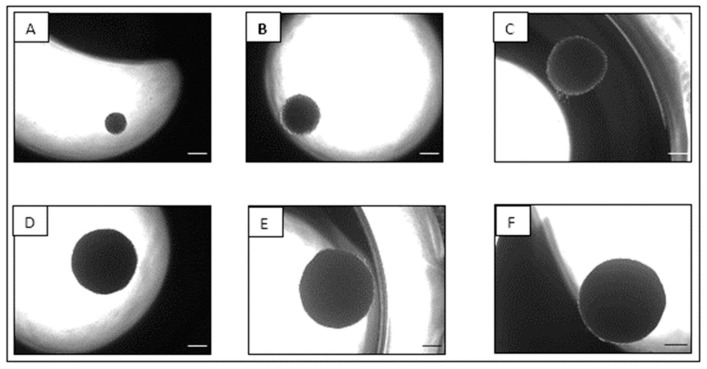
Photomicrographs of the NCI-H69AR spheroids cultured in ClinoReactors™ as observed on (**A**) day 7, (**B**) day 9, (**C**) day 13, (**D**) day 17, (**E**) day 21 and (**F**) day 25 of culture (4× magnification; scale bars = 200 µm).

**Figure 2:**
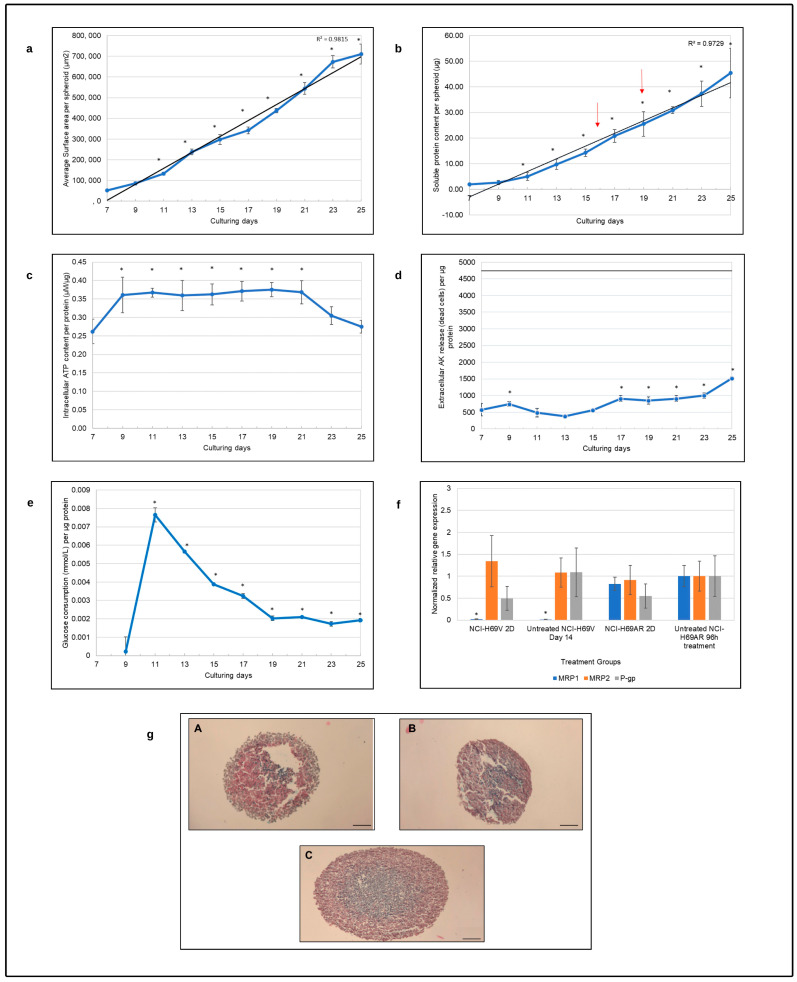
Characterization of the NCI-H69AR spheroid model as a function of time in terms of (**a**) average planar surface area per spheroid (µm^2^), (**b**) soluble protein content per spheroid (µg), (**c**) intracellular adenosine triphosphate content per soluble protein (µM/µg), (**d**) extracellular adenylate kinase release per µg protein and (**e**) approximate glucose consumption (mmol/L) per µg protein. The red arrows indicate a reduction in spheroids per ClinoReactor™; the solid black horizontal line in graph (**d**) indicates the maximum extracellular adenylate kinase per µg protein that can be released. Error bars = standard deviation (n = 6). * Statistically significant difference, *p* ≤ 0.05 (one-way ANOVA study followed by Tukey B posthoc test). (**f**) Normalized relative *MRP1*, *MRP2* and *P-gp* gene expression. Data were normalized to the untreated NCI-H69AR control group after 96 h of treatment (n = 3; error bars = standard deviation); * = statistically significant; *p* < 0.01 (one-way ANOVA followed by Bonferroni posthoc test for comparison with the untreated control). (**g**) Histological photomicrographs of NCI-H69AR spheroids stained with HE and alcian blue on (**A**) day 13, (**B**) day 16 and (**C**) day 19 of culture (scale bar = 100 µm).

Photomicrographs shown in Figure 1 illustrate the physical development and morphology of the NCI-H69AR spheroids from day 7 to the end of the 25-day characterization period. It can be observed from Figure 1 that the spheroids increased in size and became more compact structures over time. The planar surface area of the NCI-H69AR spheroids was plotted as a function of time, shown in Figure 2a as an indicator of spheroid growth and size [49]. The NCI-H69AR spheroids increased in size in an almost linear manner (R^2^ = 0.9815) over time, indicating consistent growth.

#### 3.1.2. Soluble Protein Content

On day 7, 180 spheroids were placed into each experimental ClinoReactor™ and the soluble protein content was measured every second day. The soluble protein content per spheroid as a function of time over the 25 days is shown in Figure 2b. The protein content increased consistently (R^2^ = 0.9729) throughout the characterization period, which is indicative of growth, although, between day 7 and day 9, the increase was somewhat slower. This could have been due to handling during the sorting of the spheroids on day 7, causing a disturbance to the spheroids. On day 16, the number of spheroids per ClinoReactor™ were reduced to 140 spheroids, and on day 19 again reduced to 120 spheroids, as indicated by the red arrows in Figure 2b.

#### 3.1.3. Intracellular ATP Content

The NCI-H69AR spheroid intracellular ATP content was expressed in terms of protein content to correct for both spheroid growth and population size (see Figure 2c). From day 7 to day 9, there was a significant increase in ATP per protein (*p* ≤ 0.05), and the ATP per protein thereafter remained very consistent until day 21. This suggested that the model was viable and metabolically stable during this time. From day 21, the extracellular ATP per protein decreased, which could suggest a gradual loss of viability during this time, although the protein content continued to increase in the same period of time.

#### 3.1.4. Extracellular Adenylate Kinase Content

The extracellular AK per protein released from the NCI-H69AR spheroids plotted as a function of time is shown in Figure 2d. AK release per protein increased slightly between days 7 and 9, suggesting that the handling of the spheroids on day 7 potentially stressed and damaged the cells somewhat. Although, this could also have been a result of the adaptation of the cells from a 2D to a 3D environment. AK release decreased to similar levels as those observed on day 7 between days 9 and 15, but from day 17, a statistically significant increase (*p* ≤ 0.05) was observed. These values, however, were then stable until day 23, when they increased. This increase correlated with the reduction in ATP/protein values in the same period, which could suggest a slight loss in viability. However, these AK levels were still significantly lower than the maximum AK per protein achievable in the ClinoReactors™ (horizontal line on the graph), suggesting that minimal cell death occurred during the 25-day characterization period.

#### 3.1.5. Approximate Glucose Consumption

Approximate glucose consumption can be indicative of cellular metabolic activity during spheroid growth. The glucose content of the fresh growth medium was measured as 21.4 mmol/L on average (n = 6). The glucose content in the spent medium of each ClinoReactor™ was subtracted from the average glucose content of the fresh culture medium, determining the total approximate glucose consumption per ClinoReactor™. Glucose consumption was normalized relative to the total protein content in each ClinoReactor™ (number of spheroids x protein content per spheroid) to determine the glucose consumption per protein to correct for the biomass present (Figure 2e). On day 7, when spheroids were sorted, a fresh growth medium containing 3 µg/mL of ascorbic acid was added to each ClinoReactor™. This was taken as the initial glucose value present in each ClinoReactor™. The glucose consumption per protein on day 9 was low, followed by a sharp increase until day 11. From day 11 to day 19, the glucose consumption per protein decreased gradually and then stabilized. At all sampling points from day 11, the glucose consumption per protein was statistically significantly higher when compared to day 9. The data indicate that the spheroids were metabolically active during the 25 days of characterization.

#### 3.1.6. Efflux Transporter Relative Gene Expression

In Figure 2f, the relative *MRP1*, *MRP2* and *P-gp* gene expression is illustrated for both the 2D and 3D cultured NCI-H69AR cells as well as for previously established chemosensitive NCI-H69V spheroids [41]. These results confirmed that, as stated in the literature [50], MRP1 is hyper-expressed by NCI-H69AR cells compared to the NCI-H69V cells when cultured in 2D. It is shown in the literature that H69AR and H69 small-cell lung cancer cells express MRP2 [51,52]. *MRP2* relative gene expression, however, was slightly reduced in the NCI-H69AR cells, as shown in Figure 2f.

When the NCI-H69V cells were cultured as spheroids, *MRP2* expression was reduced and *P-gp* expression increased [41]. A slight increase in *MRP1* expression was observed in the NCI-H69AR spheroid model when compared to the 2D cells, while *P-gp* expression almost doubled. This could result in the NCI-H69AR spheroid model being resistant to even more treatments than its 2D counterpart. However, *MRP2* and *P-gp* relative gene expression was very similar in both the NCI-H69V and NCI-H69AR spheroid cultures. The NCI-H69AR functional spheroids still hyper-expressed *MRP1* when compared to the NCI-H69V spheroids.

#### 3.1.7. Histology

Figure 2g shows photomicrographs of hematoxylin and eosin (HE)- and alcian blue-stained NCI-H69AR spheroids sampled on days 13, 16 and 19 to observe the organization as well as the morphology of cells in the spheroids. Alcian blue staining was performed to visualize the presence of mucin in the spheroids. As shown in Figure 2g, the NCI-H69AR SCLC tumor cells consisted of small oval, short and spindle-shaped cells. Hyperchromatic nuclei were present. These tumor cells grew in sheets of small cells that were tightly packed, and intratumoral necrosis was present, as shown in Figure 2(gA,gB), more prominently, as in Figure 2(gC). The presence of mucin was observed in all three images. As the spheroids matured, the mucin was situated more in the center of the spheroids than at the outer rim of the spheroid, as was observed on day 13.

### 3.2. Evaluation of the NHI-H69AR Spheroid Model Treatment Reactivity

For the NCI-H69AR spheroid model to be deemed suitable for anticancer treatment activity screening, its reactivity to standard chemotherapeutic treatments was evaluated. Three standard drugs, irinotecan hydrochloride, paclitaxel and cisplatin, were used as model drugs. These treatments were administrated to the spheroid groups at clinical doses used to treat SCLC patients for a 96 h period whereafter a 96 h recovery period was incorporated. All the data were normalized relative to the untreated control group. The same spheroid initiation and maintenance procedures used for the characterization of the model were used for the treatment evaluation. Spheroids were prepared for treatment on day 7 of culturing and treatment was initiated on day 9 (0 h). Spheroids were sampled every 24 h over the 96 h period. The drug-containing medium in the presence of 3 µg/mL of ascorbic acid was exchanged every 24 h after sampling and the drug dosage was adjusted daily according to the remaining biomass per ClinoReactor™. After the 96 h treatment, treatment ceased and the spheroids were cultured in a fresh culture medium for a further 96 h. This was to evaluate the ability of the spheroids to recover after treatment and determine if the effect of the chemotherapeutic drugs was sustained. Table 1 below indicates the gene expression levels before the data were normalized.

#### 3.2.1. Irinotecan Treatment Reactivity

**Figure 3:**
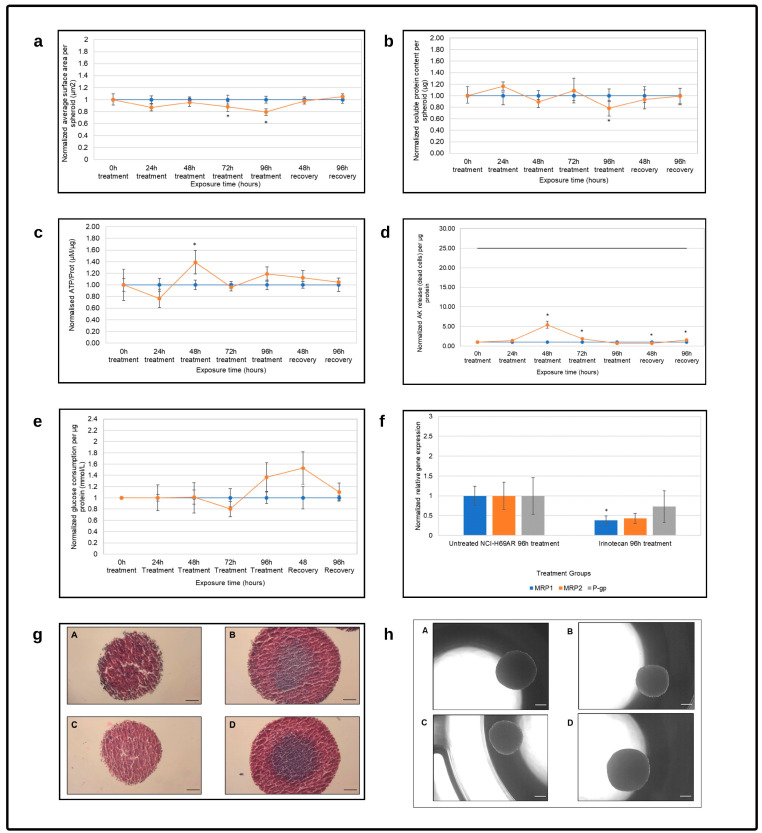
Evaluation of the NHI-H69AR spheroid model reactivity following irinotecan treatment as a function of time in terms of (**a**) average planar surface area per spheroid (µm^2^), (**b**) soluble protein content per spheroid (µg), (**c**) intracellular adenosine triphosphate content per soluble protein (µM/µg), (**d**) extracellular adenylate kinase release per µg protein and (**e**) approximate glucose consumption (mmol/L) per µg protein. The blue line represents the normalized untreated control group, the orange line represents the irinotecan treated group. The solid black horizontal line in graph (**d**) indicates the maximum extracellular adenylate kinase per µg protein that can be released. Error bars = standard deviation (n = 6). * Statistically significant difference; *p* ≤ 0.05 (HLM method). (**f**) Normalized relative *MRP1*, *MRP2* and *P-gp* gene expression. Data were normalized to the untreated NCI-H69AR control group after 96 h of treatment (n = 3; error bars = standard deviation); * = statistically significant; *p* < 0.01 (one-way ANOVA followed by Bonferroni posthoc test for comparison with the untreated control). (**g**) Histological photomicrographs of NCI-H69AR spheroids stained with HE and alcian blue after (**A**) 96 h of exposure in the untreated group, (**B**) 96 h of the recovery period in the untreated group, (**C**) 96 h of treatment with irinotecan and (**D**) 96 h of recovery after irinotecan treatment (scale bar = 100 µm). (**h**) Photomicrographs of the (**A**) untreated group after 96 h of treatment, (**B**) untreated group after a 96 h recovery period, (**C**) irinotecan group after 96 h of treatment and (**D**) irinotecan group after 96 h of recovery (scale bar = 200 µm).

Treatment with irinotecan for 24 h and 48 h had no significant effect on spheroid growth, as indicated by the planar surface area and soluble protein content. The irinotecan treatment group did significantly decrease the planar surface area after 72 h and 96 h of treatment, as shown in Figure 3a, as well as the soluble protein content levels after 96 h of treatment, as shown in Figure 3b. When the administration of irinotecan was terminated, however, the spheroid growth recovered to such an extent that the planar surface area and soluble protein content were similar to those of pre-treatment levels, with no statistically significant differences.

The normalized ATP/protein (µM/µg), plotted as a function of time, is shown in Figure 3c. Irinotecan treatment initially slightly reduced intracellular ATP levels, but a statistically significant increase was observed after 48 h of treatment when compared to the normalized untreated control. There was a second but much smaller increase after 96 h of treatment as well. The ATP levels consistently decreased during the recovery period to pre-treatment levels. Extracellular AK per protein showed a corresponding spike following 48 h of exposure to irinotecan. This simultaneous significant increase in ATP and AK could indicate apoptosis, although this observation was not prolonged and cells appeared to recover following the cessation of treatment [43]. Compared to the maximum cell death possible at each time point, as indicated by the solid black line in Figure 3d, the extent of cell death was also very modest. This observation was supported by the slight decrease in glucose consumption at the 72 h time point, suggesting that there were fewer metabolically active cells present. This was followed by a sudden increase in glucose consumption at the 96 h treatment and 48 h recovery time points. It is possible that less resistant cells initially succumbed through apoptosis, while remaining cells either were resistant or increased resistance to the treatment and, through increased repair mechanisms, recovered, and glucose consumption returned to pretreatment concentrations [53,54].

Visually, as observed in Figure 3h, the irinotecan treatment had no significant effect on the structure and size of the NCI-H69AR spheroids and they remained compact and round with smooth edges. Histological evaluation following HE and alcian blue staining of the NCI-H69AR spheroids (Figure 3g) showed mucus to be present at the outer rim of the untreated spheroid at time point 96 h. Small cells without prominent nuclei were visible. The spheroids consisted of diffuse sheets of small cells, with necrosis and stroma visible inside the spheroids. Irinotecan-treated spheroids also consisted of small malignant cells with observable necrosis, but mucus was limited. Following 96 h recovery the untreated and the treated spheroids consisted of dense sheets of small cells with scant cytoplasm, inconspicuous or absent nucleoli, and mucus in the center of the spheroids. Crush artifacts caused the smearing of nuclear chromatin. Some necrosis was visible in both groups.

The relative gene expression of *MRP1*, *MRP2* and *P-gp* decreased following irinotecan treatment compared to the untreated group. Although, this decrease was only significant for *MRP1*. The dysregulation of efflux transporters plays a crucial role in tumor formation, improves tumor progression and causes poor clinical outcomes in cancer patients. Although certain drug molecules are substrates for a specific efflux transporter, the specific efflux transporter can be expressed to a lesser extent when compared to the expression of other efflux transporters present in the tumor, indicating that the physiological role of ABC transporters contributes to tumor biology and, in turn, patient prognosis, rather than in certain cases of drug-efflux ability [55].

#### 3.2.2. Paclitaxel Treatment Reactivity

**Figure 4:**
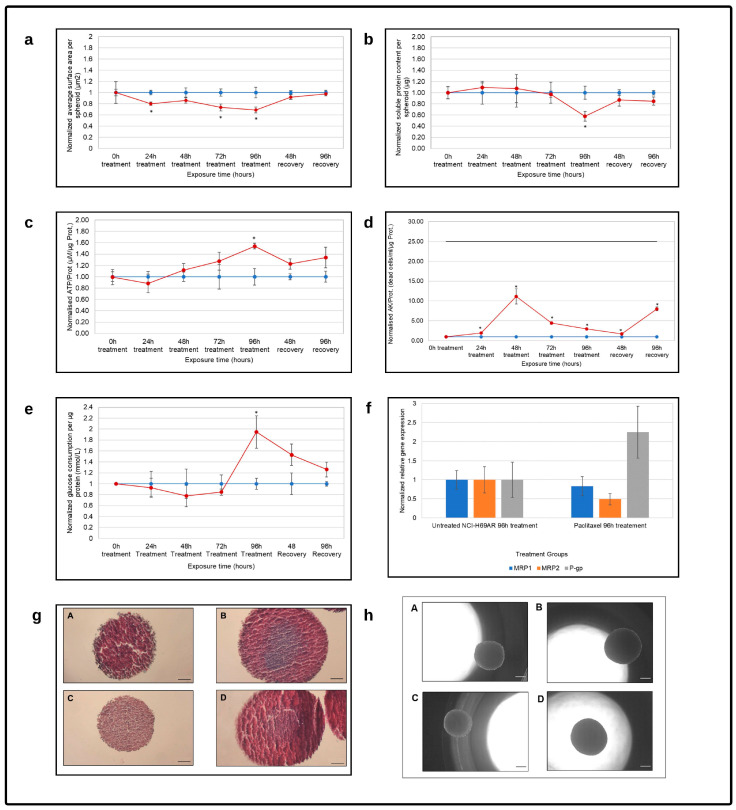
Evaluation of the NHI-H69AR spheroid model reactivity following paclitaxel treatment as a function of time in terms of (**a**) average planar surface area per spheroid (µm^2^), (**b**) soluble protein content per spheroid (µg), (**c**) intracellular adenosine triphosphate content per soluble protein (µM/µg), (**d**) extracellular adenylate kinase release per µg protein and (**e**) approximate glucose consumption (mmol/L) per µg protein. The blue line represents the normalized untreated control group, the red line represents the paclitaxel treated group. The solid black horizontal line in graph (**d**) indicates the maximum extracellular adenylate kinase per µg protein that can be released. Error bars = standard deviation (n = 6). * Statistically significant difference; *p* ≤ 0.05 (HLM method). (**f**) Normalized relative *MRP1*, *MRP2* and *P-gp* gene expression. Data were normalized to the untreated NCI-H69AR control group after 96 h of treatment (n = 3; error bars = standard deviation); * = statistically significant; *p* < 0.01 (one-way ANOVA followed by Bonferroni posthoc test for comparison with the untreated control). (**g**) Histological photomicrographs of NCI-H69AR spheroids stained with HE and alcian blue after (**A**) 96 h of exposure time in the untreated group, (**B**) 96 h of the recovery period in the untreated group, (**C**) 96 h of treatment with paclitaxel and (**D**) 96 h of recovery after paclitaxel treatment (scale bar = 100 µm). (**h**) Photomicrographs of the (**A**) untreated group after 96 h of treatment, (**B**) untreated group after a 96 h recovery period, (**C**) paclitaxel treatment group after 96 h of treatment and (**D**) paclitaxel treatment group after 96 h of recovery (scale bar = 200 µm).

Paclitaxel exposure significantly and consistently reduced the planar surface area throughout treatment with the chemotherapeutic drug, as shown in Figure 4a, with only a slight increase at the 48 h time point. Following removal of the paclitaxel, the spheroids recovered to such an extent that the planar surface area was similar to that of the untreated control group after 96 h of recovery. In contrast, the soluble protein content was slightly elevated for the first 48 h of treatment, as shown in Figure 4b. Thereafter, it decreased consistently to reach significantly reduced levels after 96 h of exposure to paclitaxel when compared to the untreated control. Following recovery, the soluble protein content of the paclitaxel-treated spheroids recovered but remained lower than that of the untreated control, although not statistically significantly so. Paclitaxel clearly influenced the growth of the NHI-H69AR spheroids, but the effect was limited and reversible.

After 24 h of treatment with paclitaxel, the ATP/protein (µM/µg) decreased slightly, as shown in Figure 4c. It then linearly increased over time to a statistically significant level after 96 h of treatment. There is a direct correlation between drug resistance and ATP production in cancer cells. Cancer cells high in ATP are the most aggressive cells, with stem-like properties, which enhance multi-drug resistance and increase the capacity for cell migration, voluntary metastasis and invasion. It is believed that these cells can withstand the effect of a hostile micro-environment as well as the effect of conventional chemotherapy. This allows these cells to naturally select for survival due to their high ATP content, which promotes tumor relapse and distant metastasis [56]. During the recovery period, the ATP/protein (µM/µg) decreased again but remained slightly elevated compared to that of the untreated control. AK per protein was significantly elevated following exposure to paclitaxel for the duration of the treatment, with the highest peaks at 48 h of treatment and 96 h of recovery. However, these were still less than half the maximum achievable cell deaths at those time points. The lack of an ATP/protein peak at 48 h could indicate necroses due to exposure to paclitaxel and, although the effects were much more pronounced than with the irinotecan treatment, it still appeared to be mostly reversible. This was again supported by the approximate glucose consumption, shown in Figure 4e, which decreased to its lowest point after 48 h of treatment, followed by a statistically significant increase after 96 h of treatment. But, again, glucose consumption decreased during recovery to similar concentrations as that of the untreated control.

Figure 4h illustrates the effect of the paclitaxel on the size and structure of the spheroids and, although 96 h of treatment clearly reduced the spheroid size compared to the untreated spheroids, the structure remained compact and round with smooth edges. As stated, the treated spheroids recovered after the removal of the paclitaxel, and the spheroid sizes were comparable to those of the untreated control. Histological evaluation, as shown in Figure 4g, showed that mucus was limited in the paclitaxel-treated spheroids, with necrosis visible in all groups. Following recovery, cells in the paclitaxel-treated spheroids were much less compact. Necrosis was visible, but to a lesser extent than in the untreated group, and mucus was interwoven between the small cells in the spheroid.

Paclitaxel administration decreased *MRP1* and *MRP2* relative gene expression in the H69AR spheroids, but significantly induced *P-gp* expression. In two studies by Synold et al. and Smit et al., it was shown that paclitaxel could induce *P-gp* expression [57,58].

#### 3.2.3. Cisplatin Treatment Reactivity

**Figure 5:**
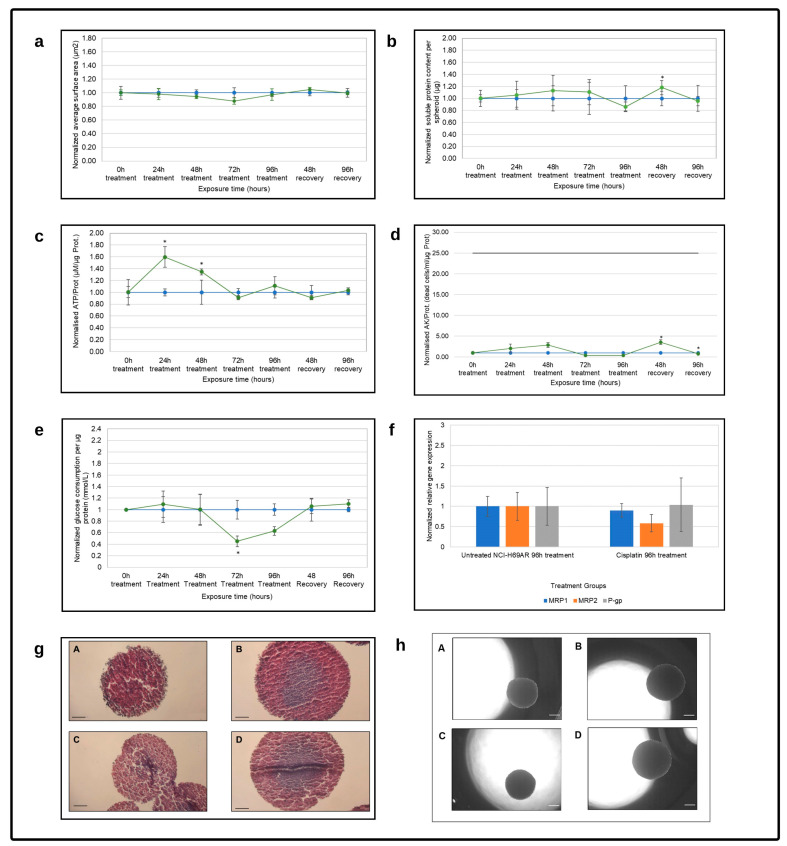
Evaluation of the NHI-H69AR spheroid model reactivity following cisplatin treatment as a function of time in terms of (**a**) average planar surface area per spheroid (µm^2^), (**b**) soluble protein content per spheroid (µg), (**c**) intracellular adenosine triphosphate content per soluble protein (µM/µg), (**d**) extracellular adenylate kinase release per µg protein and (**e**) approximate glucose consumption (mmol/L) per µg protein. The blue line represents the normalized untreated control group, and the green line represents the cisplatin treated group. The solid black horizontal line in graph (**d**) indicates the maximum extracellular adenylate kinase per µg protein that can be released. Error bars = standard deviation (n = 6). * Statistically significant difference; *p* ≤ 0.05 (HLM method). (**f**) Normalized relative *MRP1*, *MRP2* and *P-gp* gene expression. Data were normalized to the untreated NCI-H69AR control group after 96 h of treatment (n = 3; error bars = standard deviation); * = statistically significant; *p* < 0.01 (one-way ANOVA followed by Bonferroni posthoc test for comparison with the untreated control). (**g**) Histological photomicrographs of NCI-H69AR spheroids stained with HE and alcian blue after (**A**) 96 h of exposure time in the untreated group, (**B**) 96 h of recovery period in the untreated group, (**C**) 96 h of treatment with cisplatin and (**D**) 96 h of recovery after cisplatin treatment (scale bar = 100 µm). (**h**) Photomicrographs of the (**A**) untreated group after 96 h of treatment, (**B**) untreated group after a 96 h recovery period, (**C**) cisplatin treatment group after 96 h of treatment and (**D**) cisplatin treatment group after 96 h of recovery (scale bar = 200 µm).

Cisplatin had no significant effect on the planar surface area of the NHI-H69AR spheroids throughout the experiment, as shown in Figure 5a. Similarly, the soluble protein content remained relatively stable throughout the treatment period, except for a slight decrease after 96 h of exposure. Although a statistically significant increase in soluble protein content was observed after 48 h of recovery, it decreased again to levels similar to those of the untreated control group. NHI-H69AR spheroid size and growth, therefore, appeared unaffected by exposure to cisplatin.

ATP/protein (µM/µg) were significantly elevated in the first 48 h of cisplatin treatment, as shown in Figure 5c. It then returned to pre-treatment levels for the duration of the study. Extracellular AK per protein remained negligible during treatment with cisplatin, as shown in Figure 5d, but was significantly increased during the recovery period. However, relative to the maximum achievable cell death, this increase was very insignificant. This apparent lack of influence of cisplatin on cell viability and death contrasts with a significant decrease in approximate glucose consumption per protein that was observed after 72 h of treatment, as shown in Figure 5e. But, this decrease was again reversed to pre-treatment levels by the end of the recovery period.

Treatment also did not appear to affect the spheroid shape and size, as observed in Figure 5h. Histologically, mucus started to develop in the center of the cisplatin-treated spheroids after 96 h, with extensive necrosis observed. After 96 h of recovery, the necrosis was more pronounced in the cisplatin-treated spheroids than in any of the other treatment groups, as observed in Figure 5g. This is consistent with the increased AK/protein without simultaneous ATP/protein increases, which is typical of necrosis.

Cisplatin had negligible effects on *MRP1* and *P-gp* relative gene expression, with a slight decrease in *MRP2* expression. Cisplatin resistance is associated with MRP2 expression. MRP2 transport glutathione–platinum conjugates from within tumor cells and plays an important role in platinum resistance in the treatment of small-cell lung cancer [59].

## 4. Discussion

The NCI-H69AR spheroids were maintained and characterized for 25 days to observe and evaluate their growth and viability. Planar surface is a parameter used to indicate spheroid growth and size and is used to relate the physical spheroid size with the protein content [60]. The protein values of the NCI-H69AR spheroids as well as the planar surface area increased over time in an almost linear manner. Both the soluble protein content and the planar surface area over time showed consistent continuous growth of the spheroid model.

Intracellular ATP content is a widely accepted method to indirectly determine the number of viable cells in 2D and 3D cell cultures [61]. When the cells lose membrane integrity and cell viability, the remaining ATP is depleted by ATPases. Intracellular ATP concentrations are also influenced by cellular stress factors, physiological changes due to treatment and cell viability [62]. Adenylate kinase is a protein situated in the intermembrane spaces of the mitochondria, helping to maintain metabolic equilibrium, and plays a key role in ATP energy transfer from the mitochondria used to supply energy for cellular processes [43,63]. As soon as membrane integrity is lost due to cell death or direct damage to the cells, AK is released from within the intermembrane spaces of the mitochondria, indicating a loss of membrane integrity [41,43,64]. Due to the close relationship between AK and ATP and the effects on cell viability, both cell growth and cell death must be taken into consideration. During the characterization process of the NCI-H69AR spheroid model, the measured ATP and AK values were normalized per microgram of protein. This accounted for the change in biomass due to the growth or removal of spheroids. AK as well as ATP values were relatively stable between days 9 and 21, suggesting that metabolic equilibrium was achieved in this period. It also indicates that this would be the optimal experimental window for cell viability-related investigations. AK values increased from day 23, while ATP values started to decrease from day 21, possibly suggesting a loss of viability of the model.

Histologically and cytologically, SCLC is described as diffuse sheets of small round, oval or spindle-shaped cells that contain scant cytoplasm and unostentatious or absent nucleoli with salt and pepper-like chromatin. Frequent and extensive necrosis is usually present, and nuclear chromatin smearing or crushed artifacts are present inside these tumors. Extensive necrosis is due to a high mitotic rate [65,66]. Histological staining of NCI-H69AR spheroids correlates with these typical characteristics of SCLC tumors, which indicates that this 3D model mimics their tissue of origin. On day 15, the pH levels of the culture medium decreased to below the physiological pH of 7. Although the number of spheroids per ClinoReactor™ were reduced to 140 spheroids on day 16 and 120 spheroids on day 19, this did not result in a significant change in the low pH values. Since SCLC is known to produce lactic acid through aerobic glycolysis, these tumor cells rapidly remove excess lactic acid into the tumor microenvironment to prevent the accumulation of lactic acid in the cells and prevent metabolic inhibition. Another mechanism by which the acidity in the tumor microenvironment is caused is an outflow of CO_2_. The CO_2_ binds with H_2_O, resulting in the production of numerous amounts of H^+^ and bicarbonate ions. The acidity of the tumor micro-environment improves angiogenic invasion and the metastases of tumors. Acidity also has a direct correlation to drug resistance to radiation and chemotherapy [67]. The histological similarity of the spheroid model to that of in vivo tumors, combined with the increased production of mucin and the lowering of the culture medium pH, all highlight the physiological relevance of the NCI-H69AR mini-tumor model.

NCI-H69AR cells are known for their elevated expression of MRP1 (ABCC1), an ABC transporter, resulting in their resistance to various chemotherapeutic agents [68]. The hyperexpression of *MRP1* was confirmed through qPCR analysis and compared to the drug-sensitive NCI-H69V cells. It was also shown that this hyperexpression remained when NCI-H69AR cells were cultured as functional spheroids. In addition, *P-gp* relative gene expression was also slightly elevated, which could potentially result in the increased resistance of this mini-tumor model.

During the evaluation of the treatment reactivity of the NCI-H69AR spheroid model, three chemotherapeutic drugs (irinotecan, paclitaxel and cisplatin) were administrated at the clinical doses used to treat SCLC in humans. The spheroids were treated for a period of 96 h whereafter treatment was terminated, and a 96 h recovery period followed to determine spheroid recovery. In Table 2, the major findings following 96 h of treatment and 96 h of recovery are summarized.

The NCI-H69AR spheroids showed reactivity to some extent to all treatments. Since irinotecan is a known substrate for MRP1-mediated efflux, it was expected that the MRP1-hyperexpressive NCI-H69AR spheroids would have significant resistance to treatment with this therapeutic. The lack of significant and prolonged reduction in growth and viability of the model following exposure to irinotecan, with complete recovery after cessation of the treatment, supports the resistance of the mini-tumor model to MRP1-substrate chemotherapeutic drugs.

When considering all the measured parameters, paclitaxel had the most pronounced cytotoxic effect on the NCI-H69AR functional spheroids during the 96 h treatment. This was combined with a significant reduction in spheroid size and growth and significant changes in ATP/protein, AK/protein and approximate glucose consumption levels. During the 96 h recovery period, the effects of all three treatments appeared to be reversed or overcome, and the spheroids recovered to functionality and size similar to that of the untreated spheroids. This apparent resistance to treatment could be related to drug efflux since the qPCR results following 96 h of treatment with paclitaxel indicated that paclitaxel-induced the expression of the *P-gp* (*ABCB1*) efflux transporter, possibly facilitating its own efflux. Other studies also showed that the administration of paclitaxel could enhance *P-gp* expression [57,58]. Another factor that could have influenced the activity of paclitaxel is the overlapping resistance of P-gp and MRP1 efflux transporters. The structural resemblance of these two efflux transporters is only 19%, but MRP1 efflux transporters often facilitate the efflux of P-gp effluxed drug molecules [69].

Cisplatin had very minimal effects on spheroid growth and viability during treatment, although some necrosis was apparent during the recovery phase. This again suggests at least some level of resistance of the model to this treatment. This is possibly due to the MRP1 hyperexpression in the model since, in a study by Fang et al., it was observed that the overexpression of MRP1 led to cisplatin resistance [70]. ABC transporters require ATP hydrolysis as an energy resource to eliminate certain substrates, and glutathione is required to transport certain substances in cells during their detoxifying mechanisms. Glutathione binds to cisplatin through its reactive -tiol group, which prevents the drug from reacting with the DNA inside a cell, preventing DNA damage. Glutathione is a substrate for a simultaneous reaction with cisplatin prior to MRP-mediated transport [71]. The conjunction of the platinum–glutathione bindings leads to the inactivation of cisplatin as well as an increase in cisplatin solubility [28]. Glutathione disulfate and glutathione are transported by MRP1 and MRP2, and there is a direct correlation between glutathione and ABC transporters. This suggests that glutathione can induce conformation changes that induce MRP-mediated efflux [25,71].

The NCI-H69AR functional spheroids established in this study produced significant levels of mucin, contrary to the cells when cultured in 2D. Mucins can influence the processes of tumor progression, invasion and metastasis. Mucins are, therefore, an important factor in tumor cell survival and protection against the immune response of the affected host. An increase in mucin production has been observed in lung cancer [72], and secretory mucins act as a protective mechanism against exogenous cytotoxicity in tumor cells and increase the aggressiveness of a tumor [73]. Secretory mucins can cause web formations and heavily glycosylated membrane-bound mucins. Muc 1, for example, has an extraordinary size. These two mechanisms can limit intracellular drug absorption as well as the accessibility of the plasma membrane of a tumor cell. MRP1 is directly associated with mucin 1 (Muc 1) production since Muc 1 promotes MRP1 resistance [74]. Muc 1 has also been associated with cisplatin and paclitaxel resistance in lung cancer [75,76]. When considering the histological evaluations in this study, it was observed that these NCI-H69AR spheroids produce mucus. This could contribute to the multidrug resistance mechanisms of the NCI-H69AR mini-tumor model.

## 5. Conclusions

The established NCI-H69AR spheroid model is viable and functional for at least 21 days and can be used for cytotoxicity experiments from day 9 of culturing. Some morphological characteristics correlate with SCLC in humans, and properties such as increasing acidity in its environment and mucus production highlight the physiological relevance of this mini-tumor model.

This model was shown to be resistant to three known chemotherapeutic drugs, and the possible mechanisms of resistance include the hyperexpression of ABC efflux transporters, the production of mucus, glutathione binding or a combination of these factors. The NCI-H69AR mini-tumor model has, therefore, been shown to be fit for purpose to be used for the future screening of novel chemotherapeutic drugs, instead of traditional flat cultures. This will enable more accurate and physiologically relevant results, reducing false positives. The model can potentially also be used to study mechanisms of multidrug resistance in SCLC. Three-dimensional cell culture methods can be divided into two main classes, namely, anchorage-independent and anchorage-dependent. Anchorage-independent methods can further be divided into four subclasses, which include low-adhesion plates, hanging drop methods, magnetic levitation and spinner bioreactors. Anchorage-dependent methods are divided into scaffolds and hydrogels. In Table 3, the main advantages and disadvantages of these methods are summarized. By making use of a CelVivo ClinoStar^®^ system for the development of the NCI-H69AR mini-tumor drug-resistant model, many advantages were achieved. This included the highly consistent volume of the cell chamber allowing very precise and consistent treatment per biomass, similar to in vivo and clinical studies. The recovery of cells after initiation is possible using this approach. No specialized medium was required for the culturing of the mini-tumors. Temperature and gas exchange was highly controlled in the ClinoReactors^®^ due to the minimized opening of the incubation system. Easy medium exchange was possible and numerous assays could be performed due to the high spheroid yield. Highly reproducible and uniform mini-tumors were formed, and sampling was easy [77].

Three-dimensional models are known to typically be more resistant to treatment than their two-dimensional counterparts, and they resemble the in vivo environment better. In a study by Rossouw et al., the activity of selected anti-cancer compounds in multidrug-resistant cell cultures was evaluated in a conventional 2D cell culture model. In their findings, it was concluded that an NCI-H69AR cell culture was reactive to paclitaxel treatment, with a resistance ratio of 1.7 [86]. To be considered resistant, the ratio should be higher than 2. In our novel 3D model of the cell line, paclitaxel did not have a significant effect on cell viability and the model appeared to be resistant to the chemotherapeutic. This could have been because *P-gp* was hyperexpressed when compared to the conventional 2D cell culture model. *P-gp* expression also increased in the 3D model following paclitaxel treatment, which could be one of the mechanisms of paclitaxel resistance acquisition. These results highlight the need for models that represent the in vivo setting as a drug screening tool for pre-clinical studies and new drug molecule development to avoid the overestimation of activity.

In another study, Van der Merwe et al. developed a chemosensitive mini-tumor SCLC model in ClinoReactors^®^ using the NCI-H69V cell line [41]. The chemosensitive model was also qualified through the evaluation of its reactivity to irinotecan treatment. Comparing the results obtained by Van der Merwe et al. with the irinotecan treatment results obtained in this study highlights the resistance to irinotecan treatment of the newly developed model. Furthermore, the gene expression profiles confirm that the resistant model hyperexpresses MRP1 compared to the NCI-H69V model. The potential parallel use of these two models has great potential in the simultaneous screening of new treatment options for both chemosensitive as well as drug-resistant SCLC.

## Figures and Tables

**Table 1:** Relative gene expression of untreated NCI-H69AR spheroids after 13 days in culture, prior to normalization.

**Untreated NCI-H69AR 96** **h Treatment Gene Expression Values**					
**Relative *MRP1* Gene** **Expression**		**Relative> *MRP2* Gene** **Expression**		**Relative *P-gp* Gene** **Expression**	
1.371	±0.442	1.25	±0.504	0.151	±0.069

**Table 2:** Summary of normalized data for each treatment group following 96 h of treatment with irinotecan, paclitaxel and cisplatin and the subsequent 96 h of recovery.

	Normalized Average Surface Area		Normalized Soluble Protein Content per Spheroid		Normalized ATP/Prot. Values		Normalized AK Release per µg Protein		Normalized Glucose Consumption per µg Protein	
	96 h Treatment	96 h Recovery	96 h Treatment	96 h Recovery	96 h Treatment	96 h Recovery	96 h Treatment	96 h Recovery	96 h Treatment	96 h Recovery
Untreated control	1	1	1	1	1	1	1	1	1	1
Irinotecan	0.79	1.05	0.78	0.99	1.19	1.05	0.69	1.57	1.37	1.11
Paclitaxel	0.69	0.98	0.58	0.85	1.54	1.34	2.92	7.99	1.94	1.26
Cisplatin	0.97	1.00	0.86	0.95	1.11	1.03	0.40	0.73	0.63	1.10

**Table 3:** Advantages and disadvantages of different 3D tumor models.

	3D Culture Method	Advantages	Disadvantages	References
Anchorage-independent methods	Low-adhesion plates	Relatively easy method Low cost Reproducible Potential to study cell–cell interactions Useful in drug screening Uniform spheroid size control	Low throughput Spheroids must be transferred for analysis Low throughput production	[78,79]
	Hanging drop methods	Relatively easy method Uniform spheroid size Low cost Homogenous spheroids	Small size of spheroids No long-term culturing Cannot be used for drug testing Medium exchange is difficult Lack of extracellular matrix Labor intensive Impossible to conduct migration and invasion studies	[79,80,81]
	Magnetic levitation	High efficiency Easy method No specialized medium is required Medium can easily be exchanged	Colors the 3D constructs brown, limiting certain applications Cells adhere to the bottom of the wells Cellular behavior can be affected	[34,81]
	Spinner bioreactors	Relatively easy method Mass production Long-term culturing Medium composition is homogenous Can be used for high-throughput screening Not a labor-intensive method Stimulates metabolite transport	Specialized equipment is required High shear forces are present Un-uniform spheroids Medium exchange is difficult High volume of medium is required	[34,81,82]
	ClinoStar^®^ system	Relatively easy method Mass production Long-term culturing Medium composition is homogenous Stimulates metabolite transport Low shear forces are present Uniform spheroids Medium exchange is easy	Specialized equipment is required Fixed volume of 10 mL	[77,83]
Anchorage-dependent methods	Scaffolds	Physical strength can easily be adjusted Numerous materials can be used Mimics the in vivo environment Possible to mimic ECM presentation exactly as in the in vivo environment Possible to combine specific growth factors	Homogeneous dispersion of cells is problematic Expensive Labor intensive Possibility that scaffolds can variate	[78,79,80,81]
	Hydrogels	Resembles tissue-like flexibility Easy to supply water-soluble factors to the cells	Mechanical resistance is low	[78,79]
Other 3D methods	Microfluidic devices	Uniform spheroid formation Possible to control spheroid size Rapid spheroid formation Continuous perfusion Oxygen and nutrient distribution are uniform Constant temperature control	Expensive Labor intensive Requires specialized equipment and expertise	[80,81,84]
	3D bioprinters	Hypoxic conditions inside a spheroid Produces complex 3D architecture Intercellular interactions are possible Possible to reproduce paracrine Reproducing 3D tissue architecture is possible Possible to mimic the chemical environment inside a tumor High throughput screening is possible Can mimic the complex interactions between the tumor microenvironment and the ECM	Difficult to standardize the method Reproducibility of the interactions between the cells and the ECM is not possible Expensive consumables and equipment Difficult to position the cells Optimization of the printing resolution still needs to be improved	[84,85]

## Data Availability

Data are available upon request.
